# Adherence and Body Weight with Daily Avocado Consumption Among Latina Women of the Habitual Diet and Avocado Trial (HAT)

**DOI:** 10.3390/nu17020367

**Published:** 2025-01-20

**Authors:** Tiffany Q. Luong, Mopelola A. Adeyemo, Penny M. Kris-Etherton, Alice H. Lichtenstein, Nirupa R. Matthan, Kristina S. Petersen, David M. Reboussin, Joan Sabaté, Zhaoping Li

**Affiliations:** 1 Fielding School of Public Health, UCLA, Los Angeles, CA 90095, USA; tqluong@g.ucla.edu; 2 Division of Clinical Nutrition, Department of Medicine, David Geffen School of Medicine at University of California, Los Angeles, CA 90095, USA; madeyemo@mednet.ucla.edu; 3 Department of Nutritional Sciences, Penn State University, University Park, PA 16802, USA; pmk3@psu.edu; 4 Cardiovascular Nutrition Laboratory, Jean Mayer USDA Human Nutrition Research Center on Aging, Tufts University, Boston, MA 02111, USA; alice.lichtenstein@tufts.edu (A.H.L.); nirupa.matthan@tufts.edu (N.R.M.); kup63@psu.edu (K.S.P.); 5 Department of Biostatistics, School of Medicine, Wake Forest University, Winston-Salem, NC 27157, USA; drebouss@wakehealth.edu; 6 Center for Nutrition, Lifestyle and Disease Prevention, School of Public Health, Loma Linda University, Loma Linda, CA 92350, USA; jsabate@llu.edu

**Keywords:** avocado, body weight, randomized controlled trial, Hispanic and Latina populations, HAT

## Abstract

**Objectives:** The aim of this study was to examine the adherence, changes in weight, and, waist circumference associated with the daily consumption of a culturally preferred food, namely an avocado, among Hispanic/Latina females in the Habitual Diet and Avocado Trial (HAT). **Methods:** HAT was a multisite, randomized controlled trial conducted between 2018 and 2020. Participants in the Avocado-Supplemented Diet Group were provided with and instructed to consume one avocado/day (~2.2 servings) for 6 months; participants in the Habitual Diet Group were instructed to follow their usual diet and limit intake to ≤2 avocados/month. Avocado consumption was assessed using three random 24 h dietary recalls administered by dietitians. This analysis focused on women who self-identified as Hispanic/Latina. **Results:** Within HAT, 158 females self-identified as Hispanic/Latina (median age: 42 years, IQR: 36–54). Across the dietary recalls, the Avocado-Supplemented Group (*n* = 80) consumed 1.9–2.1 avocado servings/day; the Habitual Diet Group (*n* = 78) consumed 0.04–0.09 servings/day (*p* < 0.001). The weight and waist circumference measurements were similar between groups. Hispanic/Latina females remained adherent to daily avocado consumption for the 6-month study period, without a significant change in their body weight or waist circumference measurements. **Conclusions:** Integrating a culturally preferred food into a dietary intervention enhanced adherence amongst Latina adults, with no impact significant impact on body composition.

## 1. Introduction

Approximately two in five adults (40.3%) in the United States were obese from 2021 to 2023, according to the National Health and Nutrition Examination Survey [1]. Hispanics are more likely to have obesity and related cardiometabolic diseases, such as diabetes [2] and metabolically associated steatotic liver disease [3], compared to their non-Hispanic white counterparts. Furthermore, Hispanic women are more likely to have obesity than Hispanic men.

Given the high prevalence of cardiometabolic disease in the Latin community, effective dietary and lifestyle interventions are needed to prevent excessive weight gain, facilitate weight loss, and sustain long-term weight management tailored to this community. Because Latin adults have been shown to lose less weight compared to non-Hispanic white adults in intensive lifestyle interventions [4], culturally tailoring lifestyle interventions has been proposed as a method to improve the acceptability of lifestyle changes in minority communities [5]. Culturally tailoring lifestyle interventions may increase adherence to and the effectiveness of interventions, given the various cultural and environmental factors affecting weight loss among individuals of Latin heritage [6]. Several studies have reported a modest improvement in weight loss with culturally tailored interventions [7].

Acculturation is a well-studied sociocultural factor impacting the dietary patterns of immigrant populations. Greater acculturation has been associated with a higher intake of less healthy foods (e.g., sugar-sweetened beverages, and ultra-processed foods) and a lower intake of healthy foods (fruits, vegetables, and whole grains) among Hispanic/Latino adults [8]. The influence of acculturation on the diets of Latino adults has also been found to affect the dietary quality of youth living in the same households, thereby possibly playing a major role in the rising obesity rate.

When Mexican American participants were asked about their knowledge, priorities, and preferences regarding diet, exercise, and evidence-based strategies for behavioral change, one theme that arose was the desire for culturally familiar healthy food options [9]. One such culturally familiar food option is the avocado, which originated in the Mesoamerica area [10] and was consumed as early as 8000–7000 BC [11]. Avocados are nutrient-dense in that one 50 g serving of an avocado contains 80 kilocalories, 4.3 g of carbohydrates (including 3.4 g of fiber), 1.1 g of saturated fat, 4.9 g monounsaturated fat, and 0.9 g of polyunsaturated fat [12]. Increased monosaturated fat intake has been associated with increased satiety and an increase in the amount of fat burned [13], which likely contributes to the reduced body weight [14], waist circumference, and body fat seen with increased monounsaturated fat intake. Similarly, increased fiber intake has been associated with increased satiety [15], as well as weight loss [16] and a reduction in waist circumference. Though avocados are rich in monounsaturated fats and fiber, there are conflicting reports on the impact of avocados on measures of adiposity [17,18] and limited studies on the impact of avocados on weight loss in Latin adults.

In this study, our objective was to examine adherence to a culturally preferred food, an avocado, among Latina participants in the Habitual Diet and Avocado Trial (HAT) [12]. We also conducted exploratory analyses to examine the effects of avocado intake on measures of adiposity.

## 2. Materials and Methods

### 2.1. Study Cohort

The present study cohort consisted of a subset of the HAT population [12]. Briefly, participation in the HAT study was restricted to individuals who had an increased waist circumference (≥35 inches for women, ≥40 inches for men), were at least 25 years of age, and were not currently eating more than two avocados per month [19]. In the present study, this cohort included women who self-identified as Hispanic or Latina and were at least 25 years of age at the time of the study. The study population was restricted to non-pregnant women; women who were pregnant, lactating, or trying to get pregnant were excluded from participating in HAT.

The study procedures, including written informed consent from all participants, were approved by each center’s Institutional Review Board (IRB). Wake Forest University Health Sciences served as the Central IRB. The detailed study design has been published [19]. The trial is registered at clinicaltrials.gov (NCT 03528031).

### 2.2. Intervention

Briefly, the study was a 6-month intervention. Participants in the Habitual Diet Group were instructed to follow their usual diet. Participants were allowed to consume up to 2 avocados per month, but avocado consumption was not encouraged, and no avocados were provided during the study. Participants in the Avocado Supplemented Diet Group were provided with fresh avocados every two weeks and instructed to consume 1 avocado per day for 6 months.

### 2.3. Adherence

Adherence to the diet was assessed by 4 random 24 h dietary recalls. The 4 recalls were conducted within 0–2 weeks prior to randomization, at 8 weeks, at 16 weeks, and 26 weeks post-randomization. Trained research dietitians conducted telephone recalls using the Nutrition Data System for Research (NDSR). Adherence to avocado consumption was quantified by measuring the frequency and number of avocadoes consumed in the Avocado-Supplemented Diet Group and the lack of consumption in the Habitual Diet Group. According to the NDSR, one whole large avocado (168 g) equated to roughly 2.2 servings of avocado.

### 2.4. Dietary Quality

Healthy Eating Index (HEI) scores were calculated to assess dietary quality. A maximum score of 100 suggested that the dietary quality was consistent with the ‘Dietary Guidelines for Americans’.

### 2.5. Anthropometric Measures

The participants’ weight and waist circumference were measured at 2, 12, and 26 weeks post-randomization.

### 2.6. Analyses

Descriptive statistics were performed to summarize the mean and standard deviation (SD) of continuous variables and the number and proportions for continuous variables. A *t*-test was conducted to compare means among continuous variables, whereas a Χ^2^ test was used to compare distributions among categorical variables.

## 3. Results

### 3.1. Study Cohort

**Figure 1:**
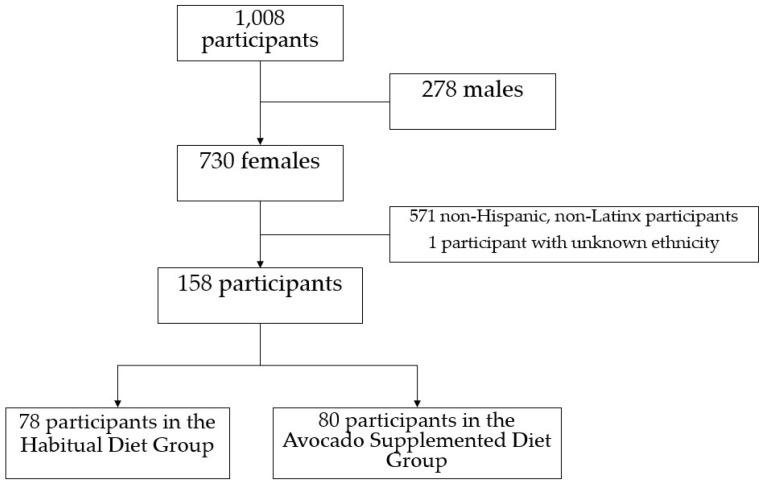
Cohort selection.

Among the 1008 participants in the HAT trial, 158 self-identified as Hispanic or Latina and were at least 25 years of age at the time of the study (Figure 1). Of these 158 women, 78 were assigned to the Habitual Diet Group and 80 were assigned to the Avocado-Supplemented Diet Group. Demographic and other characteristics measured at baseline were not significantly different between groups (Table 1).

### 3.2. Compliance

Across the four 24 h dietary recalls, participants in the Habitual Diet Group consumed an average of 0.04–0.09 servings of avocado (Table 2). Prior to randomization, participants in the Avocado-Supplemented Diet Group consumed an average of 0.1 servings of avocado on the day of recall. Across the three dietary recalls conducted at 8, 16, and 26 weeks post-randomization, participants in the Avocado-Supplemented Diet Group consumed an average of 1.9–2.1 servings of avocado on the day of recall. At all post-randomization dietary recalls, the Avocado-Supplemented Diet Group was adherent with avocado consumption.

### 3.3. Dietary Quality

Among participants in the Habitual Diet Group, the average HEI score was 56.3 (SD 11.9) at baseline, and scores were between 55.5 and 57.2 during follow-up (Table 2). Among participants in the Avocado-Supplemented Diet Group, dietary quality was similar to that of the Habitual Diet Group (*p* = 0.07). At 8 and 16 weeks post-randomization, dietary quality was higher among the Avocado-Supplemented Diet Group (*p* < 0.001 and *p* = 0.02, respectively). At the last recall, dietary quality was similar between groups (*p* = 0.06).

### 3.4. Anthropometric Measures

Table 3 presents the anthropometric and laboratory measures across follow-up and according to the randomization arm. The baseline measures for weight and waist circumference were similar between groups (*p* = 1.00 and *p* = 0.78, respectively). At the end of follow-up, the average weights were 86.3 kg (SD 13.5) in the Habitual Diet Group and 87.2 kg (SD 18.6) in the Avocado-Supplemented Diet Group (*p* = 0.73). Waist circumference was similar between groups at the end of follow-up (*p* = 0.80).

## 4. Discussion

Latinos are underrepresented in clinical trials and often have poor adherence to healthy diets when they participate in clinical trials [20]. In this study, we evaluated the impact of using a culturally preferred food, avocados, on adherence to a dietary intervention among Latina adults, as well as its effect on measures of adiposity. In this study, we found that (1) Latina adults were adherent to the 6-month dietary intervention in both the Avocado-Supplemented Diet Group (≥1 avocado per day), consuming between 1.9 and 2.1 avocados per day, and the Habitual Diet Group (<2 avocados per month), consuming between 0.04 and 0.09 avocados per day. We also found that (2) changes in anthropometric measures were similar within and between groups over the intervention period.

### 4.1. Adherence to Provided Foods

Our study found that Latina adults adhered to dietary recommendations over a 6-month period. The data on adherence to healthy diets in minority communities, including Latin communities, are conflicting [21,22]. While some studies report poor adherence to healthy diets amongst minority communities [21], other studies report increased adherence [22]. Social determinants of health play a significant role in food choices [22,23]. In particular, a lower socioeconomic status and food insecurity are associated with poorer dietary quality. Latin communities are twice as likely to be food insecure (16% of Latin households) in comparison to non-Hispanic white households (8% of non-Hispanic white households). This disparity is likely to contribute to the poorer dietary quality seen amongst Latin households. In our study, participants were provided with the food needed for the intervention rather than having to purchase it themselves, which may have contributed to the increased adherence. The provision of food in nutrition clinical trials helps address food insecurity by mitigating financial barriers to healthy eating [24]. Several studies have found that providing food not only improves adherence to dietary interventions, but also retention in nutrition clinical trials [25]. Jenikins et al. compared the impact of providing dietary advice and/or food provisions to 919 overweight adults on cardiometabolic risk factors [26]. They found that at 6 months, there was 91% retention in the group provided with food; however, the retention rate was 67% for those without food provided (*p* < 0.0001). Furthermore, there was a modest improvement in the intake of fruit and whole grains amongst the group provided with food compared to the group that was not provided with food. Similarly, in the multi-center PREDIMED clinical trials, participants who were provided with food had increased adherence to the mediterranean diet after 1 year [25]. This suggests that future studies including dietary interventions in diverse communities should consider providing food to enhance adherence and retention. Additionally, studies should address negative social determinants of health as a potential barrier to adherence to dietary interventions.

### 4.2. Adherence to Culturally Preferred Foods

The increased adherence to dietary intervention in our study may also be due to the use of a culturally preferred food choice. We evaluated adherence to an avocado, as avocados have been shown to be preferred amongst Latin communities [27]. When looking at the food preferences of 254 Mexican Americans in comparison to non-Hispanic white adults, Mexican Americans consumed significantly more avocados than non-Hispanic white adults. This suggests that culture plays a role in dietary choices. Several studies have shown that culturally tailoring dietary interventions enhances adherence [28,29,30]. Thus, in conjunction with these studies, our findings suggest that including culturally preferred foods in dietary interventions may improve the adherence of Latin communities in nutrition clinical trials in the future.

### 4.3. Impact of Avocado Intake on Anthropometric Measures

The results regarding the impact of avocado intake on weight and waist circumference are conflicting [31,32]. Our study did not reveal significant changes in the participants’ weight or waist circumference after 6 months of increased avocado intake, which is similar to other studies in Latin communities [31,32]. However, these findings are conflicting, as other studies have shown less weight gain with increased avocado intake [17]. Thus, additional studies are needed to evaluate the impact of avocado intake on weight and waist circumference. Still, increased avocado intake has been associated with an improvement in other important cardiometabolic measures, such as improved insulin resistance in those with type 2 diabetes [33,34] and a lower incidence of hypertension [35]. Thus, increasing avocado intake may be a weight-neutral intervention that improves other cardiometabolic diseases for which Latin communities are at an increased risk.

### 4.4. Limitations

One limitation of our study is that participants who identified as Hispanic or Latina were not further categorized by their country of origin. Culturally preferred foods may vary within ethnic groups based on both country of origin and geographic location, which may have impacted adherence to the dietary intervention. However, due to the number of participants included in this subset analysis, we were unable to evaluate if adherence to the diet varied by country of origin or geographic region. Another limitation of our study is that due to the small sample size of this sub-group analysis, we were unable to adjust for potential confounders in the between-group analyses of HEI scores. This limitation may impact the generalizability of our findings. Therefore, further studies are needed to evaluate the impact of incorporating preferred foods on dietary quality in Latin communities. We used 24 h dietary recall to evaluate adherence to dietary intervention. This method presents a potential limitation of our study due to the risk of recall bias, as individuals are more likely to overestimate their adherence to dietary recommendations on recall. However, the National Data System for Research, which was used for the collection of 24 h dietary recall data, has been used effectively in several diverse settings and studies [36].

## 5. Conclusions

The findings from this study suggest that incorporating culturally preferred foods into dietary interventions may improve adherence and increase the acceptability of dietary interventions in Latin communities. This is key to improving the inclusion of minority communities in nutrition clinical trials. Furthermore, the study findings highlight the importance of culturally tailoring evidence-based dietary recommendations for minority communities to improve adherence to a healthy diet in the clinical setting, ultimately improving cardiometabolic equity.

## Figures and Tables

**Table 1:** HAT Hispanic/Latina characteristics at baseline (*n* = 158).

	Habitual Diet Group *n* = 78	Intervention *n* = 80	Total *n* = 158	*p*-Value
Age				
Mean (SD)	44.1 (12.0)	44.9 (12.2)	44.5 (12.1)	0.69
Median (IQR)	41.5 (35.4, 54.2)	42.2 (35.7, 53.3)	41.8 (35.5, 53.7)	0.77
BMI				
Mean (SD)	33.0 (5.3)	32.6 (5.7)	32.8 (5.5)	0.61
Median (IQR)	32.0 (30.0, 36.0)	31.0 (28.5, 35.5)	32.0 (29.0, 36.0)	0.40
BMI Category, *n* (%)				0.39
Normal Weight (18.5–<25 kg/m^2^)	2 (3)	4 (5)	6 (3.8)	
Overweight (25.0–<30 kg/m^2^)	16 (21)	22 (28)	38 (24.1)	
Obesity (≥30 kg/m^2^)	60 (77)	54 (68)	114 (72.2)	
Race				0.85
American Indian	1 (1)	2 (3)	3 (1.9)	
Black or African American	2 (3)	5 (6)	7 (4.4)	
White	54 (69)	54 (68)	108 (68.4)	
Other Race	17 (22)	14 (18)	31 (19.6)	
Multiple Race	3 (4)	4 (5)	7 (4.4)	
Unknown Race	1 (1)	1 (1)	2 (1.3)	
Waist Circumference				
Mean (SD)	107.5 (11.2)	107.2 (11.5)	107.3 (11.3)	0.87
Median (IQR)	105.0 (98.0, 114.0)	105.0 (99.0, 115.0)	105.0 (99.0, 115.0)	0.79
Living Situation				0.25
House	48 (62)	59 (74)	107 (67.7)	
Apartment	25 (32)	13 (23)	43 (27.2)	
Other	5 (6)	3 (4)	8 (5.1)	
Education				0.55
None	4 (5)	7 (9)	11 (7.0)	
Vocational School	14 (18)	11 (14)	25 (15.8)	
Community or Junior College	32 (41)	24 (30)	56 (35.4)	
Four-Year College	20 (26)	23 (29)	43 (27.2)	
Graduate School	6 (8)	11 (14)	17 (10.8)	
Professional School	1 (1)	3 (4)	4 (2.5)	
No Answer	1 (1)	1 (1)	2 (1.3)	
Retired				0.16
Yes	4 (5)	9 (11)	13 (8.2)	
No	74 (95)	71 (89)	145 (91.8)	
Smoking History				0.37
Never Smoker	64 (82)	65 (81)	129 (81.6)	
Current Smoker	0 (0)	1 (1)	1 (0.6)	
Former Smoker	14 (18)	12 (15)	26 (16.5)	
Unknown	0 (0)	2 (3)	2 (1.3)	

SD indicates standard deviation; IQR, interquarterile range; and BMI, body mass index.

**Table 2:** Compliance with avocado consumption and Health Eating Index scores at 0–2 weeks prior to randomization, and 8, 16, and 26weeks post-randomization (*n* = 158).

		Habitual Diet Group *n* = 78				Avocado-Supplemented Diet Group *n* = 80			
		0–2 Weeks Prior	8 Weeks Post	16 Weeks Post	26 Weeks Post	0–2 Weeks Prior	8 Weeks Post	16 Weeks Post	26 Weeks Post
Servings of Avocado ^a^	-	0.07 (0.25)	0.09 (0.33)	0.04 (0.12)	0.04 (0.14)	0.11 (0.37)	2.10 (0.63) ***	1.94 (0.80) ***	1.90 (1.04) ***
Components	Maximum Points	0–2 Weeks Prior	8 Weeks Post	16 Weeks Post	26 Weeks Post	0–2 Weeks Prior	8 Weeks Post	16 Weeks Post	26 Weeks Post
Total HEI Score	100	56.3 (11.9)	56.5 (16.1)	55.5 (15.1)	57.2 (15.9)	52.3 (15.5)	64.6 (12.4)	60.9 (13.6)	61.6 (13)
Adequacy									
Total Fruits	5	1.9 (2.0)	2.2 (2.1)	2.0 (2.0)	1.7 (2.0)	1.4 (1.8)	4.3 (1.2)	4.1 (1.4)	3.9 (1.6)
Whole Fruits	5	2.1 (2.1)	2.4 (2.3)	2.3 (2.2)	1.9 (2.1)	1.7 (2.1)	4.7 (0.9)	4.6 (1.3)	4.4 (1.6)
Total Vegetables	5	3.3 (1.6)	3.7 (1.6)	3.5 (1.5)	3.4 (1.7)	3.5 (1.7)	3.1 (1.7)	2.9 (1.6)	3.0 (1.7)
Greens and Beans	5	2.2 (2.3)	2.6 (2.4)	2.2 (2.4)	2.7 (2.4)	2.5 (2.4)	2.5 (2.4)	1.9 (2.3)	2.2 (2.3)
Whole Grains	10	5.3 (4.3)	4.4 (4.4)	3.5 (4.5)	4.7 (4.3)	3.4 (4.2)	4.2 (4.2)	3.3 (4.0)	3.7 (4.2)
Dairy	5	5.0 (3.7)	3.8 (3.5)	4.5 (3.6)	4.5 (3.5)	4.6 (3.2)	4.4 (3.4)	4.4 (3.3)	4.3 (3.5)
Total Protein Foods	5	4.3 (1.3)	4.6 (0.9)	4.6 (1.0)	4.6 (1.1)	4.5 (1.1)	4.1 (1.3)	4.0 (1.5)	4.2 (1.4)
Seafood and Plant Protein	10	2.6 (2.3)	2.8 (2.4)	2.4 (2.4)	2.9 (2.3)	2.9 (2.3)	2.1 (2.3)	2.3 (2.4)	2.1 (2.4)
Fatty Acids	10	5.1 (4.0)	5.4 (3.8)	5.8 (3.8)	5.4 (3.8)	4.7 (3.9)	7.5 (3.3)	7.2 (3.2)	7.6 (2.8)
Moderation									
Refined Grains	10	7.5 (3.4)	7.3 (3.5)	6.9 (3.7)	7.2 (3.7)	6.3 (3.8)	7.4 (3.4)	7.3 (3.0)	7.1 (3.6)
Sodium	10	4.2 (3.8)	3.8 (3.6)	4.1 (3.7)	5.0 (3.7)	4.3 (4.0)	6.2 (3.4)	5.3 (3.6)	5.4 (3.9)
Added Sugars	10	7.8 (2.6)	7.6 (2.8)	8.1 (2.9)	8.0 (2.5)	7.4 (2.9)	8.7 (1.9)	8.4 (2.4)	8.2 (2.8)
Saturated Fats	10	5.1 (3.7)	5.8 (3.6)	5.7 (3.4)	5.2 (3.5)	5.0 (3.5)	5.4 (3.7)	5.1 (3.5)	5.5 (3.6)

^a^ One avocado is 2.2 servings. *** *p* < 0.001 compared to the Habitual Diet Group.

**Table 3:** Anthropometric and laboratory measures at 2, 12, and 26 weeks post-randomization (*n* = 158).

	Habitual Diet Group *n* = 78			Avocado-Supplemented Diet Group *n* = 80			*p*-Value		
Measure/Weeks	**2**	**12**	**26**	**2**	**12**	**26**	**2**	**12**	**26**
Mean Weight (SD), kg	86.2 (13.3)	86.2 (12.8)	86.3 (13.5)	86.2 (18.0)	86.9 (18.7)	87.2 (18.6)	1.00	0.78	0.73
Mean Waist Circumference (SD), cm	106.9 (10.6)	106.8 (10.8)	106.1 (12.1)	106.4 (11.4)	106.4 (12.2)	106.6 (12.9)	0.78	0.83	0.80

## Data Availability

Data is contained within the article.
